# Spatial transcriptomic analysis of the mouse brain following chronic social defeat stress

**DOI:** 10.1002/EXP.20220133

**Published:** 2023-10-13

**Authors:** Ting Wang, Zhihong Song, Xin Zhao, Yan Wu, Liying Wu, Abbas Haghparast, Haitao Wu

**Affiliations:** ^1^ Department of Neurobiology Beijing Institute of Basic Medical Sciences Beijing China; ^2^ Neuroscience Research Center, School of Medicine Shahid Beheshti University of Medical Sciences Tehran Iran; ^3^ Key Laboratory of Neuroregeneration, Co‐innovation Center of Neuroregeneration Nantong University Nantong China; ^4^ Chinese Institute for Brain Research Beijing China

**Keywords:** chronic social defeat stress, depression, gene expression, spatial transcriptomics

## Abstract

Depression is a highly prevalent and disabling mental disorder, involving numerous genetic changes that are associated with abnormal functions in multiple regions of the brain. However, there is little transcriptomic‐wide characterization of chronic social defeat stress (CSDS) to comprehensively compare the transcriptional changes in multiple brain regions. Spatial transcriptomics (ST) was used to reveal the spatial difference of gene expression in the control, resilient (RES) and susceptible (SUS) mouse brains, and annotated eight anatomical brain regions and six cell types. The gene expression profiles uncovered that CSDS leads to gene synchrony changes in different brain regions. Then it was identified that inhibitory neurons and synaptic functions in multiple regions were primarily affected by CSDS. The brain regions Hippocampus (HIP), Isocortex, and Amygdala (AMY) present more pronounced transcriptional changes in genes associated with depressive psychiatric disorders than other regions. Signalling communication between these three brain regions may play a critical role in susceptibility to CSDS. Taken together, this study provides important new insights into CSDS susceptibility at the ST level, which offers a new approach for understanding and treating depression.

## Graphical abstract

In this study, Wang et al. explored the potential pathogenesis of chronic social defeat stress (CSDS)‐induced depressive‐like behaviour in mice using 10× Visium spatial transcriptomics (ST) technique. Many of the depression‐related gene changes were identified and more pronounced in the hippocampus (HIP), cortex (Isocortex), and amygdala (AMY) in susceptible (SUS) mice compared to control and resilient (RES) mice. In particular, compared to other cell types, inhibitory interneurons, and synaptic function were most significantly affected in multiple brain regions following CSDS.

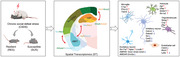

## 1 INTRODUCTION

Mental disorders pose huge economic challenges to the whole society. Promoting mental health and well‐being has been incorporated into the United Nations 2015–30 sustainable development goals.^[^ ^1^ ^]^ Among them, depression is a highly common and disabling mental disorder, which not only causes a lot of human suffering and health loss, but also affects economic output. Additionally, the global pandemic of COVID‐19 has brought complex social impacts and led to a significant increase in mental illness.^[^ ^2^ ^]^ Stress is the main cause of depression and other emotional disorders. Individuals have different responses to stress, and only some people will have depressive symptoms. The chronic social defeat stress (CSDS) mouse model is a behaviourally effective rodent model, which shows a long‐term physiological and behavioural phenotype similar to depression and anxiety.^[^ ^3^, ^4^ ^]^ It can mimic the individual differences in human stress responses and is closest to the pressure of human social environment. CSDS can effectively simulate the main symptoms of human mental diseases.^[^ ^5^, ^6^ ^]^

Depression usually involves changes in many genes and is related to abnormal functions in multiple brain regions.^[^ ^7^, ^8^, ^9^ ^]^ Many brain structures, neurotransmitters, hormones, and substances may participate in the development of Major Depressive Disorder (MDD).^[^ ^10^ ^]^ Previous studies have used coexpression analysis in depressed human postmortem brain or mouse stress models to describe interesting changes in gene interaction networks in a single brain region.^[^ ^7^, ^11^ ^]^ However, there are few transcriptome‐wide characterizations to identify the transcriptional characteristics of multiple brain regions in depression.

Although single‐cell RNA sequencing (scRNAseq) has depicted comprehensive brain atlas of different species in single‐cell resolution which can characterize the global expression pattern of each brain region, it cannot analyze the spatial pattern of gene expression. The transcriptional profiles of a single cell will be affected by their localization in the tissue.^[^ ^12^ ^]^ Location information is crucial for exerting tissue‐specific functions, maintaining tissue homeostasis, and responding to external cues or interference.^[^ ^13^ ^]^ More importantly, the spatial organization and function of the brain are fundamentally linked, especially in human cortical tissue, different cortical cells show variable gene expression patterns and physiological morphology.^[^ ^14^ ^]^ Spatial transcriptomics (ST),^[^ ^15^, ^16^, ^17^, ^18^, ^19^ ^]^ a non‐targeted technology, is rapidly becoming an extension of scRNAseq. It has the potential to analyze gene expression with single‐cell resolution while maintaining the cell composition in the tissue. ST technology enables researchers to better understand cell interactions and heterogeneity, and to have an in‐depth understanding of complex biological processes that cannot be achieved by traditional sequencing technology.^[^ ^20^ ^]^ Up to now, this technology has been applied to the study of neuroscience, providing new biological insights into the development of the central nervous system and the mechanism of central nervous system disorders, including molecular atlas of the adult mouse brain,^[^ ^18^ ^]^ Alzheimer's disease,^[^ ^16^, ^21^ ^]^ and amyotrophic lateral sclerosis.^[^ ^22^ ^]^

Given the complicated transcriptional characteristics of multiple brain regions in depression, we used ST to gain new insights into the spatially resolved transcriptome‐wide characterizations. In this study, CSDS mouse model was established to understand the differences in individual responses to stress and the biological principles of susceptibility changes. First, we sought to define anatomical brain regions and cell types by using well‐known markers in control, resilient, and susceptible mice respectively. We identified orchestrated gene expression changes of multiple brain regions, three easily affected brain regions (HIP, Isocortex, AMY) and inhibitory neurons which were more pronounced in depression. Second, those brain regions trend to interact among them in depression by analyzing correlation between expression profiles and cell‐cell communication. Finally, we defined genes that mediated the interaction among these three brain regions. Together, this study provides new insights into spatial gene expression patterns in a mouse model of CSDS, which may offer new avenues for understanding and treating depression. To our knowledge, the study is the first to use ST to identify the spatially molecular characterizations of multiple brain regions in depression.

## 2 MATERIAL AND METHODS

### 2.1 Animals

Adult male C57BL/6 (8–10 weeks) mice and male CD‐1 mice (4 months) were purchased from SPF (Beijing) Biotechnology Co., Ltd. Mice were placed in a specific pathogen‐free, standard animal facility with controllable temperature and humidity. They were raised in groups under 12 h light and 12 h dark cycle, and they could freely obtain food and water. All experiments with animals were performed in conformity with the protocols approved by the Institutional Animal Care and Use Committee of Beijing Institute of Basic Medical Sciences (approval No.SYXK 2019‐0004 as of September 2020).

### 2.2 Chronic social defeat stress mouse model

CD‐1 mice used in CSDS attacks were screened according to the methods reported in the literature.^[^ ^23^ ^]^ Before the experiment, 4–5 C57BL/6 mice per cage were fed for 1 week to adapt to the environment. CSDS experiment time is 9:00 AM–10:00 AM every morning. C57BL/6 mice were placed in the CD‐1 mouse cage for 10 days, attacked continuously for 5–10 min, and then isolated with a porous transparent acrylic plate for 24 h. During this period, the mice were subjected to continuous psychological pressure. CD‐1 mice were randomly replaced every day to avoid adaptation. The control group (CON) was C57BL/6 mice of the same week old. After contacting non‐aggressive C57BL/6 mice for 5–10 min every day, they were isolated with the same plate for 24 h, which was similar to the operation of the experimental group. After 10 days, the mice were taken out and fed in a single cage, and the social interaction test was carried out within 24 h.

### 2.3 Social interaction test

Mice in CSDS stress group or control group were placed in an open field (41 cm × 41 cm), with a metal mesh cage (diameter 8 cm) on one side. In the first stage, C57BL/6 mice were placed in an open field for 2.5 min when there was no target in the metal mesh cage; In the second stage, put a strange CD‐1 target mouse in the metal cage, and then put the C57BL/6 mouse back in the open field for 2.5 min. During the experiment, ANY‐maze software was used to record the social interaction area (16 cm in diameter) and social avoidance area (8 cm × 8 cm). The social interaction ratio (SI ratio) was obtained by dividing the time spent in the interaction area when the target exists by the time spent in the interaction area when the target does not exist. Social avoidance varies widely among individual mice. In the experiment, the mice with SI ratio lower than 1 during the observation period were susceptible, and the mice with SI ratio higher than 1 were resilient.

### 2.4 Brain sections for Visium spatial transcriptomics

At 1.5 h after the last stress exposure to CSDS, the brain tissue was isolated aseptically, and carefully removed blood and other contamination with ice cold sterile Hank's Balanced Salt Solution (HBSS, without calcium and magnesium chloride; 480Gibco 14175‐095). Brain tissues were immediately embedded in Optimal Cutting Compound (OCT) media and frozen on dry ice. The tissue blocks were sectioned on the cryostat at 10 μm thickness (bregma2.0–2.1) at −10°C using Leica Cryostat CM1950 cryostat and mounted on Visium Gene Expression Slides Capture Area (2000233, 10X Genomics), which were stored at −80°C before proceeding the Visium spatial gene expression experiment. The following experiments were detected with Agilent Bioanalyzer and the RNA values of the tissues were greater than 8.

### 2.5 Tissue staining and imaging for Visium spatial transcriptomics

Fixing and staining brain tissue sections according to the Visium Spatial Gene Expression User Guide (CG000239 Rev B, 10X Genomics) or Visium Spatial Tissue Optimization User Guide (CG000238 Rev A, 10X Genomics). Tissue sections were fixed in methanol (−20°C pre‐cold) for 30 min. Isopropyl alcohol was incubated at room temperature for 1 min. Slides were air‐dried. 1 mL Hematoxylin uniformly covered all tissue sections on the slides, and slides incubated at room temperature for 7 min. Water washing two times (Each immersion is 1 s). 1 mL Bluing Buffer was added, and all tissue sections were evenly covered and incubated for 2 min at room temperature, and washed with water for 1 s. Add 1 mL Eosin Mix to cover all tissue sections evenly, incubate for 1 min at room temperature, and wash with water for 1 s. Air dried the tissue. Brightfield histology images were taken using 20× objective (Zeiss) on a Pannoramic MIDI. Raw images were stitched together using 3DHISTECH and exported as .tiff files with low‐ and high‐resolution settings. For tissue optimization experiments, fluorescent images were taken with 3DHISTECH using a 20× objective and 60 ms exposure time.

### 2.6 Permeabilization and reverse transcription

Brain tissue was permeabilized for 18 min, incubated with 70 μm permeabilization enzyme on thermocycler adaptor at 37°C. After removing the permeabilization enzyme with a straw and washing with 100 μL 0.1×SSC buffer, Master Mix was added to each well to initiate reverse transcription 53°C for 45 min.

### 2.7 Spatial library preparation and sequencing

The library was constructed according to the Visium Spatial Gene Expression User Guide (CG000239). They were loaded at 300 pM and sequenced on a NovaSeq 6000 System (Illumina) using a NovaSeq S4 Reagent Kit (200 cycles, catalog no. 20027466, Illumina). Sequencing was performed using the following read protocol: read 1, 28 cycles; i7 index read,10 cycles; i5 index read, 10 cycles; and read 2, 91 cycles.

### 2.8 Sequencing data analysis and visualization

Raw FASTQ files and images were processed by ST Pipeline.^[^ ^24^ ^]^ After removed low quality reads, kept reads were mapped to the mouse genome (mm10). The count matrix tables were read into R (version 3.6.3), keep them paired with the low‐resolution histology images for visualization purposes, and further processed using the Seurat R package (version 3.0.0/4.0.6). For quality control, we removed genes with less than 10 reads or expressed in fewer than 2 spots observed, or low‐quality spots (<200 UMI counts). Then gene expressions were normalized by the SCTransform function in Seurat.

Use the SpatialFeaturePlot function in Seurat (version 3.0.0) to generate normalized spatial feature expression plots. According to the hematoxylin and eosin (H&E) images and Allen brain atlas, spot clusters representing the same regions were divided into 15 groups of anatomical regions using marker genes, which were then used for downstream analysis. PCA was then performed on significantly variable genes, and the first 15 PCs were selected as input for clustering and Umap, based on manual inspection of a PC variance plot. Clustering was performed using the default method from the Seurat package, with resolution parameter set to 1.2. Marker genes for each brain region were identified using the FindMarkers function provided in the Seurat R package, The FindMarkers function runs with the default parameters and uses a non‐parametric Wilcoxon rank sum test.

### 2.9 Correlation analysis

To identify the trends of expression changes in different brain regions after CSDS, we used gene correlation matrix to describe it. Pearson correlation was calculated according to the gene expression matrix in each brain region. The dimension was reduced by PCA, and the first two principal components were selected to show the correlation of brain regions using the ggplot2 package in R.

### 2.10 Overlap of gene expression profiles in CSDS versus control

Rank‐rank hypergeometric overlap (RRHO) is a Threshold‐free algorithm,^[^ ^25^, ^26^ ^]^ which can be used to detect and visualize overlap of genes changed in the same and opposite directions between two datasets. The genes were ranked by the log2 of fold change value between any two brain regions. Up, down, and unchanged genes were at the bottom, top, and middle of the list, respectively. By creating a heatmap using the odds ratio method,^[^ ^26^ ^]^ to show the strength, pattern, and correlation boundaries between brain regions.

### 2.11 Gene functional enrichment analysis

Differentially expressed genes (two‐sided Wilcoxon‐test, log fold‐change threshold = 0.25, *p*‐value < 0.05) of each brain region were selected and used for Gene Ontology (GO) term enrichment analysis using clusterProfiler package (version 3.18.1). To test synaptic gene enrichment, we enriched DEGs using Synaptic Gene Ontologies and annotations (SynGO: syngoportal.org) with default parameters. The enriched GO terms of interested genes were selected and visualized using ggplot2 (version 3.4.2).

### 2.12 Cell annotation

Considering that the expression profile observed at each capture location of the spatial transcriptome is a mixture of transcripts produced by one or more cells,^[^ ^27^ ^]^ to evaluate the distribution of cell types in different brain regions under different processing conditions, the convolution spatial data set was interpreted using spotlight (version 0.99.9) method. We used the published mouse brain scRNAseq data (GSE129788; 37,089 single cells)^[^ ^28^ ^]^ to annotate the six cell types (astrocytes, excitatory neurons, inhibitory neurons, oligodendrocytes, microglia, and endothelial cells) potential ratio of each spot.

### 2.13 Spatial ligand‐receptor interaction analysis

We applied NicheNet (https://github.com/saeyslab/nichenetr)^[^ ^29^ ^]^ and dsCellNet (https://github.com/songzh523/ dsCellNet)^[^ ^30^ ^]^ to research data from the spatial region microenvironment of CSDS mice. Introducing regional specificity (3 brain regions: Isocortex, HIP, AMY) in the analysis, inferring spatial region‐specific potential ligand‐receptor interactions, and showed the top 28 ligand and L–R with Circos plots (adjusted *p*‐value < 0.05, at least ligand and receptor expressed in ≥20% of cells). Ligand activity was predicted using the predict_ligand_activities function and ranked in descending order of Pearson's correlation, with the top ranked genes plotted as a heat map.

### 2.14 Immunofluorescence staining

Mouse brain sections were fixed with 4% PFA (paraformaldehyde) in PBS for 10 min and blocked with 5% fetal bovine serum (FBS) for 1 h (5% FBS, 3% Triton X‐100 in PBS). Primary antibody neurotensin (1:200, ImmunoStar 20072) was incubated overnight at 4°C. Fluorescence was detected after secondary staining with the Alexa Fluor 488‐conjugated donkey anti‐rabbit IgG (1:500, Biotium, Cat# 20015, RRID: AB_10559669) for 1 h at 37°C. All images were captured on an Olympus FV‐1200 confocal microscope (Olympus, Center Valley, PA, USA) and analyzed with Image J software (NIH, US).

### 2.15 Statistical analyses

Data analysis was performed using Prism software (GraphPad Prism version 8, GraphPad Software, Inc.). The results were expressed as the mean ± SEM. For multiple comparisons, we used the one‐way ANOVA test followed by Tukey's post‐test to specifically compare the indicated groups. The level of statistical significance was indicated as *p < 0.05, **p < 0.01, ***p < 0.001.

### 2.16 Key resources table

For more details about the deposited data, software and algorithms, and critical commercial assays, please check the key resources table.

**Table:** 

Reagent or resource	Source	Identifier
**Deposited data**		
Raw and analyzed sequencing data	This paper	GEO: GSE228394
Single‐cell transcriptomic profiling of the aging mouse brain	Ximerakis et al. 2019	GEO:GSE129788
Single‐cell RNA‐seq of mouse cerebral cortex	Zeisel, Amit et al. 2015	GEO: GSE60361
Single cell gene expression	5k_mouse_brain_CNIK_3pv3	https://www.10xgenomics.com
**Software and algorithms**		
Cell ranger versions 2.1/3.0	10X Genomics	https://www.10xgenomics.com
ST pipeline version 1.7.2	Navarro et al., 2017	https://github.com/SpatialTranscriptomicsResearch/st_pipeline
SCTransform v0.3.2	Hafemeister and Satija., 2019	https://github.com/ChristophH/sctransform
ST spot detector	Wong et al., 2018	https://github.com/SpatialTranscriptomicsResearch/st_spot_detector
Space ranger version 1.0.0	10X Genomics	https://10xgenomics.com
Seurat v4	Hao et al., 2021	https://github.com/satijalab/seurat
Seurat versions 2.3.0/3.1.3	Butler et al., 2018	https://satijalab.org/seurat/
SPOTlight	Elosua‐Bayes et al., 2021	https://github.com/MarcElosua/SPOTlight
SynGO versions 1.1	Koopmans et al., 2019	https://syngoportal.org
NicheNet	Robin et al., 2020	https://github.com/saeyslab/nichenetr
**Critical commercial assays**		
Chromium single cell 3′ gene expression solution v2	10× Genomics	Cat#PN‐120237
MiSeq reagent kit v3 (150 cycle)	Illumina	Cat#MS‐102‐3001
KAPA library quantification kit	Roche	Cat#07960298001
DNeasy blood and tissue kit	QIAGEN	Cat#69504
Visium spatial gene expression solution v1	10× Genomics	Cat#PN‐1000184
Visium spatial tissue optimization slide and reagent kit	10× Genomics	Cat#PN‐1000193

## 3 RESULTS AND DISCUSSION

### 3.1 Unsupervised clustering defines spatial distribution of expression across brain regions

**FIGURE 1:**
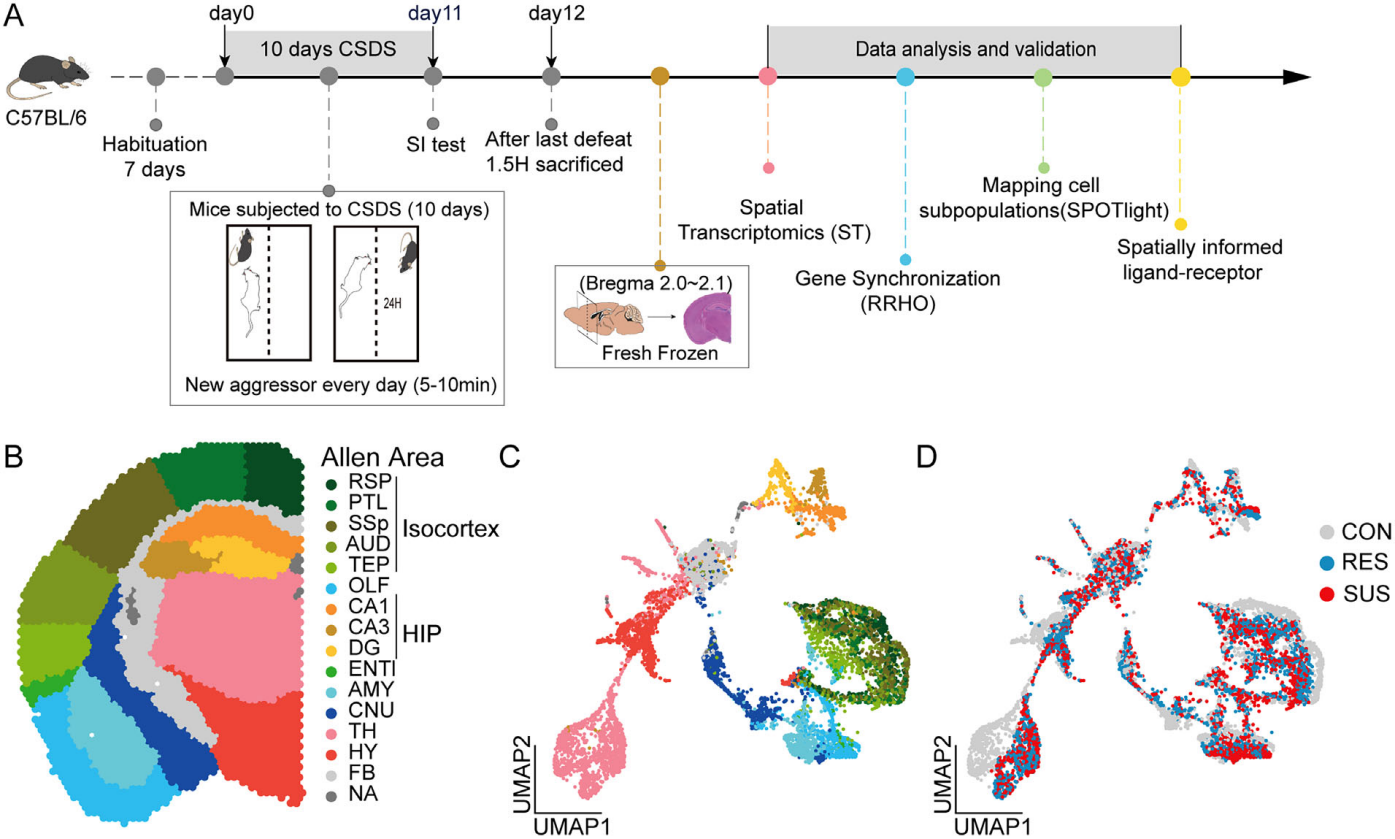
Spatial transcriptomics profiles in CSDS mouse brain. A, Schedule of behavioural experiments of CSDS and SI, tissue collection, and bioinformatics analysis. B, Brain regions were used for ST analysis referring to the Allen mouse brain atlas. C, D, UMAP plots of the transcriptomics profiles. The colour was marked according to the brain region C, same as B, or animal group D. CON, control; SUS, susceptible; RES resilient. TH, thalamus; HY, hypothalamus FB, fiber tracts; CNU, cerebral nucleus; AMY, amygdala; OLF, olfactory area; ENTl, entorhinal area; TEP, temporal association area, entorhinal area, and perirhinal area; AUD, auditory area; SSp, primary somatosensory area; PTL, posterior parietal association area; RSP, retrosplenial area. Hippocampus: Ammon's horn CA1, CA3; DG, dentate gyrus.

We studied the potential pathogenesis of depression related behaviours by using CSDS mouse model to simulate the individual variation after social defeat.^[^ ^23^ ^]^ In this model, inbred C57BL/6 mice with the same genetic background were repeatedly attacked by a larger male CD‐1 mouse every day (5–10 min) for 10 days (Figure 1A). In the experiment, mice undergoing CSDS were subjected to continuous psychological pressure of sensory interaction with the attacker through the transparent perforated partition in the shared cage. The social interaction test (SI test, Figure S1) was conducted 24 h after the last social defeat to evaluate their social interaction/avoidance behaviours against unfamiliar social goals.

After experiencing repeated intermittent psychological and physical stress, the mice were differentiated into two independent social response subgroups: resilient subgroup (RES: SI ratio > 1; continuous social interaction) and susceptible subgroup (SUS: SI ratio < 1; long‐term social avoidance). These two subgroups have been reported differently in multiple behavioural and physiological domains.^[^ ^4^ ^]^ SUS mice spend significantly less time (*p* < 0.01) in the social interaction area and significantly more time (*p* < 0.001) in corners, compared to CON mice exposed to unfamiliar CD‐1 target mice. CON mice spent most of their time in the social interaction area, while SUS mice showed a strong aversion reaction. They showed avoidance behaviour in the SI test (*p* < 0.001) (Figure S1A–C). RES mice did not show these behaviours after stress. RES mice spent comparable time in the interaction area as CON mice that did not experience CSDS, and the proportion of social interactions did not differ from CON mice.

Fresh brain tissue was collected 1.5 h after the last CSDS exposure, and three coronal sections were obtained (Bregma2.0–2.1) from the left brain of CON, RES and SUS mice by cryosections of mouse brains. Then ST sequencing was performed.^[^ ^15^, ^16^, ^17^, ^18^, ^19^ ^]^ Every coronal section contains 2875 ± 19 spots (median ± SD) (Table S1). And we detected an average of 77,470 reads, 13,481 unique molecular identifiers(median), 4,499 genes(median) per spot for the dataset (Figure S2A–D). We aligned each coronal hemisphere section with 15 anatomical brain regions (Figure 1B) defined by the Allen Mouse Brain Atlas (ABA; www.brain‐map.org), and assigned each spot a corresponding position to obtain tissue coordinates, neuroanatomical definitions, and spatially defined gene expression patterns. We visualized the spatial gene expression of the regional marker Isocortex (Lamp5), Hippocampus (HIP: Hpca), Thalamus (TH: Prkcd), Hypothalamus (HY: Gpx3), Olfactory area (OLF: SLC30a3), Cerebral nucleus (CNU: Penk), Fiber tract (FB: Mbp), Amygdala (AMY: Lypd1). The results were shown by comparing with the available ABA in situ hybridization (ISH) data (Figures S2E, S3). ISH signals correspond perfectly to the spatial gene expression in the brain atlas. After filtering out regions with very few spots, we retained eight brain regions for further analysis.

Those well‐defined brain regions clustered together according to the expression pattern using Uniform Manifold Approximation and Projection (UMAP; Figure 1C). Compared with spots of CON, these spots of RES and SUS clustered together (Figure 1D). We analyzed the differential expression of spatially highly variable genes by using Seurat, and found significantly upregulated genes in each brain region (two‐sided Wilcoxon test, log fold‐change > 0.25 and *p*‐value < 0.05) and the highest ranked genes that exhibit significant spatial dependence in their expression^[^ ^31^ ^]^ (Figure S2E).

### 3.2 Spatial identification of differently expressed genes in the CSDS brain

To investigate biological differences among different brain regions of CON, SUS and RES three independent brain sections, we calculated the gene expression correlations among eight brain regions. The results showed that the expression similarity was significantly different among brain regions, but the overall gene expression pattern was very similar among the three groups (Figure S4A). Similar expression patterns may have similar functions, and the expression similarity among Isocortex, AMY, HIP and OLF was more obvious than other regions in each group.

**FIGURE 2:**
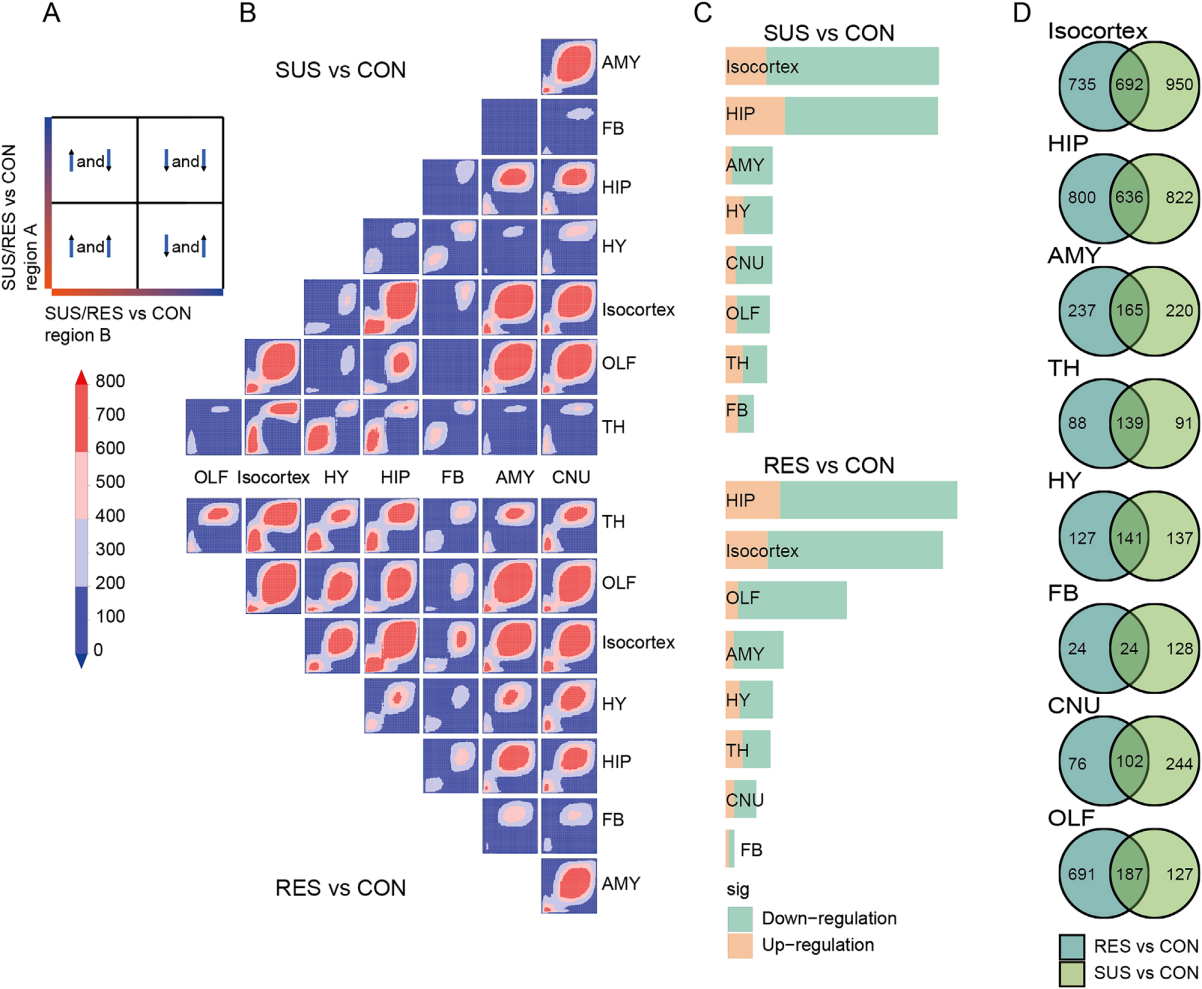
The differential Expression Patterns in RES and SUS mouse brains. A, B, RRHO maps show the comparison in gene expression between brain regions in SUS/RES vs CON (upper/lower panel). A, The extent of overlap of upregulated genes is shown in the bottom left corner, that of downregulated genes in the top right corner. B, RRHO heatmaps across brain regions. Degree of significance is depicted in colour bar on the left of RRHO maps. C, Differential gene expression analysis shows up‐ and down‐regulated genes (|Log2FC| > 0.25 and FDR < 0.05) in each brain region. D, Venn diagram displaying the number of unique and overlapping differentially expressed genes (Log2FC| > 0.25 and FDR < 0.05) in SUS/RES versus CON after CSDS.

In addition, we also confirmed that there are significant depression‐related changes in gene synchronization between the regions affected by depression, and strong changes in gene synchronization between the AMY and cingulate (CIN) regions as observed in human patients with major depression (MDD).^[^ ^8^, ^11^ ^]^ To explore the common changes of gene synchronization in brain regions of RES and SUS mice compared with CON, we used unbiased rank hypergeometric overlap (RRHO) analysis and visualized the overlapping trends between gene expression profiles of different brain regions between SUS and RES (Figure 2A,B) to calculate the common gene expression patterns across multiple datasets, which are graphically represented the intensity of patterns. RRHO test showed that compared with the CON, a large number of down‐regulated genes overlapped between different brain regions in both SUS and RES mice. The analysis of SUS showed that the transcriptional characteristics of Isocortex, HIP, AMY, OLF, and CNU regions were strongly and coordinately downregulated, and similar coordinated downregulation was found for these regional genes in the RES mice (max–log10(*p*‐value): 739–740). There were significant differences in TH and HY regions between SUS and RES mice. Signals from TH and OLF, TH and AMY, TH and CNU, HY and OLF, HY and Isocortex were stronger in RES mice than SUS mice (Table S2). In both data sets, the signals between the FB region and other brain regions were weak, which may be related to the fact that FB is mainly composed of myelinated insulating nerve fibers which play a broad connecting role in the brain.^[^ ^32^ ^]^ Although some common regulation can also be seen in many brain regions, Isocortex, AMY, HIP regions share a very obvious co‐downregulated gene pattern between SUS and RES (Figure 2B).

Next, to gain insight into stress‐related brain transcriptome characteristics for each region, SUS and RES mice were compared with CON for differential expression patterns. We compared the number of differentially expressed genes (DEGs) in each brain region in detail (Figure 2C,D). Since there are genes that are specifically expressed in brain regions or affected by different spatial patterns, it is very necessary to subdivide brain regions and define DEGs separately. When the internal subgroups of Isocortex (RSP, PTL, SSP, AUD, TEP) were finely divided, the differential genes were identified in each subregion (Figure S4B–I). The number of DEGs was highest in the Isocortex, HIP and AMY brain regions (log fold‐change > 0.25 and adjusted *p*‐value < 0.05) and lowest in the FB brain region (Figure 2C). The Venn diagram depicted the number of overlapping DEGs in each anatomical region (Figure 2D). Multi‐volcano plots of all the DEGs are shown in Figure S5A–C.

### 3.3 Spatial cell type annotation in the CSDS mouse model

**FIGURE 3:**
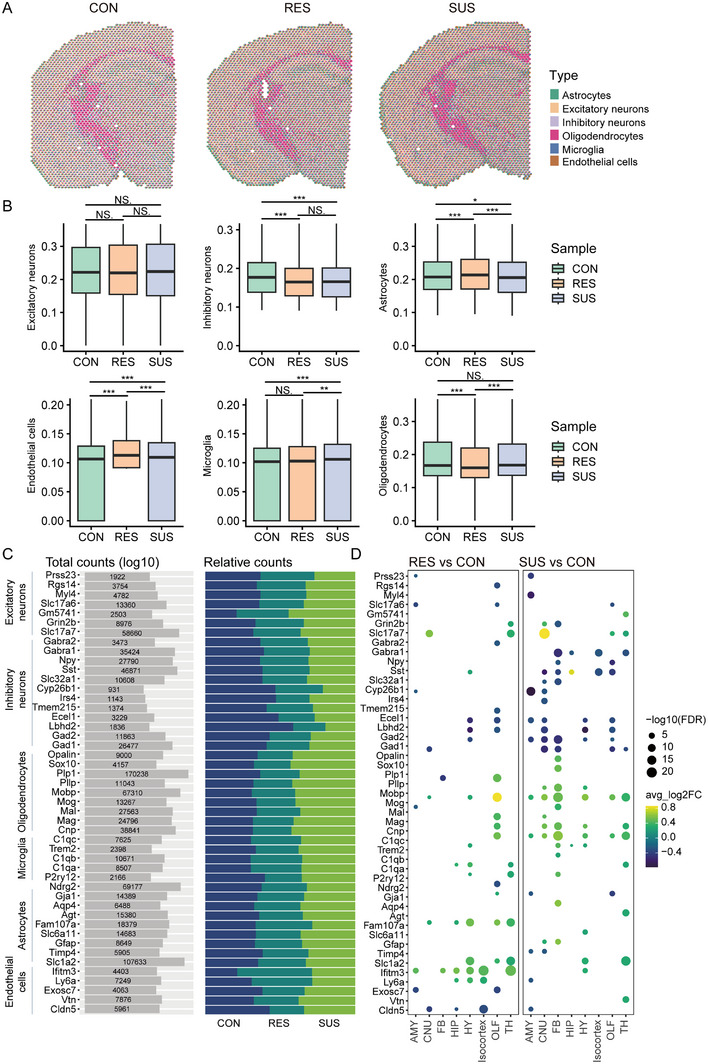
Cell type mapping on coronal mouse brain deconvoluted using Ximerakis et al.’s public scRNAseq data. A, Spatial scatter pie plot represents the proportions of the cell types from the reference atlas within capture locations. B, The proportion of predicted cell types among CON, SUS, and RES sections *p < 0.05, **p < 0.01, ***p < 0.001, ns: not significant (batch t‐test and corrected by Bonferroni test). C, Total counts show the number of marker genes detected across three coronal sections. Relative counts show the proportion of detected genes in CON, RES, SUS, respectively. D, Enrichment of marker genes in brain regions. (Benjamini–Hochberg‐corrected *p* < 0.05). The size of the balls is proportional to logged odds ratio.

Spatial resolution of gene expression profiles is essential for understanding the structure and function of brain tissues.^[^ ^33^ ^]^ Combining ST technology and scRNAseq information to deconvolute spatial data sets, which can realize in‐depth study on tissue and organ structures, clarify cell crosstalk, and track dynamic cell tracks in space. To annotate the cell types of spots, the spatial data set was deconvoluted using the scRNAseq to infer the proportion and location information of each cell type in the spatial transcriptome by SPOTlight method. Several different mouse brain scRNAseq datasets were found in public databases, and correlation analyses revealed that our experimental ST data were most similar to the scRNAseq of GSE129788 (mouse brain)^[^ ^28^ ^]^ and thus were used to provide a cellular classification basis for the spatial transcriptome data (Figure S6A). In the scRNAseq data, we just picked 24,401 single cell transcriptome data from eight young mice (2–3 months, the whole brain region after hindbrain resection) to recluster the subdivided cell types (top 20 PCs using a clustering resolution of 2.0), and identify the genes of cell‐specific markers (*p* < 0.01, two‐tailed student *t*‐test) of neurons, astrocytes, oligodendrocytes, endothelial cells, microglia cell types (Figure S6B,C).^[^ ^34^, ^35^, ^36^ ^]^ The spatial distribution of cell types in brain regions was predicted and showed enrichment of specific cell types in certain brain regions (Figures 3A, S6D–J, and S7A–H).

The deconvolution data of brain sections show the proportion of six cell types in each spot (Figure 3B, Table S3). By analyzing the abundance of each cell type, we found that changes in gene expression are mainly in the inhibitory Neurons. The SUS mice show significant differences in the areas of Isocortex, HIP, TH, FB, CNU, HY region (*p* < 0.05) compared with CON, consistent with the finding in MDD with reduced brain concentrations of the Inhibitory neurotransmitter GABA.^[^ ^37^ ^]^ In the SUS mice, GABA receptor (GABRA1) showed significant downregulation in FB, HIP, Isocortex, and TH regions (FDR < 0.05), and GABA interneurons somatostatin (SST) was significantly downregulated in Isocortex, OLF, CNU, FB, and other brain regions (FDR < 0.05) compared with CON, commonly resulting in cognitive dysfunction (Figure 3C,D). Our data is also consistent with reports of downregulation of SST mRNA expression in the dorsolateral prefrontal cortex.^[^ ^38^ ^]^ In addition, we found that genes such as Slc17a7 in excitatory neurons were highly expressed in CNU, OLF, and TH regions, C1qc in microglia were highly expressed in Isocortex and HIP regions, Cldn5 in endothelial cells were low expressed in AMY region, Slc1a2 and Slc6a1 in astrocytes were highly expressed in HY region, Cnp, and Mobp in oligodendrocytes were highly expressed in AMY, CNU and FB regions (Figure 3D, Table S4).

Understanding the cell type‐specific contribution to the pathology of depression susceptibility is important. Huang et al. concluded that CSDS consistently increased anxiety‐like behaviour in mice, both SUS and RES mice. However, impaired social interaction behaviours were only observed in susceptible mice,^[^ ^39^ ^]^ which may involve a range of physiological changes and be highly variable between individuals. Chronic stress exposure causes neuronal atrophy in cortical and limbic brain regions associated with depression, which is associated with an altered balance of excitatory and inhibitory systems.^[^ ^40^ ^]^ Chronic stress reduces the density and function of spine synapses.^[^ ^41^ ^]^ In this study, in addition to the mentioned inhibitory neurons, we also observed changes in the proportion of other cells, we observed significant changes in the proportion of excitatory neurons in SUS mice compared to the CON group, particularly in the CNU, FB, OLF, and TH brain regions. Furthermore, CSDS leads to glial and myeloid‐mediated synaptic changes. The regions where oligodendrocytes showed significant changes were AMY, CNU, and FB. Reduced oligodendrocyte density and ultrastructure were also detected in the amygdala of depressed patients.^[^ ^42^ ^]^ Decreased oligodendrocyte cell numbers, impaired OPC differentiation and deficiency of myelination, or reduction of existing sheath thickness are commonly observed in SUS animals.^[^ ^43^ ^]^ Endothelial damage and blood‐brain barrier (BBB) leakage were observed in SUS brains,^[^ ^44^ ^]^ but not in RES or CON mouse brains, as evidenced by vascular leakage of intravenously injected fluorescent tracers.^[^ ^45^ ^]^ Microglial changes were mainly observed in the Isocortex and HIP regions, while astrocyte changes were mainly observed in the AMY and OLF regions. Chronic stress induces hyperactivation and structural alterations of microglia,^[^ ^46^ ^]^ astrocyte branch morphology changes,^[^ ^47^, ^48^ ^]^ astrocytes, and microglia are important players in mediating neuroinflammatory like responses, synaptic dysfunction, and BBB impairment.^[^ ^43^ ^]^ Increased microglia‐neuron interactions have been reported in the motor cortex of susceptible mice.^[^ ^48^ ^]^ Besides that, how cross‐talk between different cells drives CSDS susceptibility is beyond the scope of this paper.

### 3.4 Stress‐induced gene dysregulation associated with synaptic function and major depression disorder

Since several brain regions, such as the prefrontal cortex, hippocampus, amygdala, thalamus, hypothalamus, have important regulatory effects on anxiety and stress response,^[^ ^49^ ^]^ the chronic activation of neural circuits in multiple brain regions may be related to the continuous changes in brain activity during chronic social stress exposure, as well as the development of anxiety and depression. We enriched the GO biological processes for DEGs in each region (Figure S8, Table S5). In these stress‐related brain regions, many identified pathways have been reported to be related to depression.^[^ ^50^ ^]^ Our results confirm that social defeat stress leads to significant dysregulation of synaptic organization, cognition, and learning or memory in multiple brain regions of RES and SUS mice. Both RES and SUS mice displayed synaptic dysfunction in the Isocortex or HIP region. Synaptic dysfunction played an important role in the pathogenesis and development of MDD.^[^ ^51^, ^52^, ^53^ ^]^ Kang et al. revealed the decrease in the expression of synaptic function related genes and the corresponding decrease in the number of synapses in dlPFC of MDD subjects.^[^ ^53^ ^]^ Our results also show that the synaptic plasticity of SUS mice was defective, as indicated by the significant dysregulation of synaptic plasticity related genes (Arc, Cplx2, Syp, Nrgn, Pmch, Calb2, Syt4) in multiple brain regions in SUS mice (Table S4), which may be due to chronic maladjustment of stress response, loss of synaptic connection and impaired synaptogenesis in these brain regions.

**FIGURE 4:**
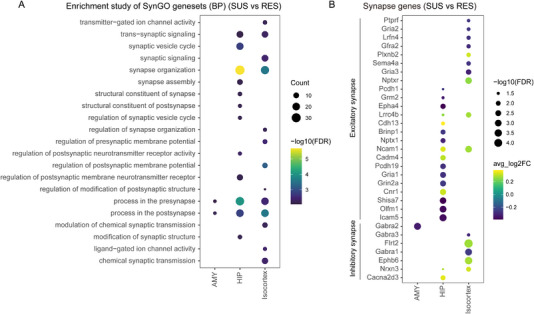
SynGO enrichment analysis identified CSDS‐induced synaptic dysfunction genes. A, Significantly enriched SynGO ontology terms using DEGs in the Isocortex, HIP, and AMY regions (FDR < 0.01, SUS vs RES). B, Significantly dysregulated synapse genes in excitatory or inhibitory synapses in SUS versus RES. Bonferroni‐adjusted *p*‐values were used to determine significance at an FDR  <  0.05.

To examine the detailed synapse‐related function of DEGs, we further compared annotated DEGs with the synaptic gene ontology database (SynGO).^[^ ^54^ ^]^ Compared with RES, 201 differential genes in SUS were identified as synaptic genes (Table S6). Compared with other brain regions, we found that genes associated with synaptic disorders were mainly enriched in Isocortex, HIP and AMY brain regions (Figure 4). Isocortex synaptic enrichment analysis identified 7 cellular components and 14 biological processes out of 78 genes, with postsynapse being the most enriched biological processes. Among the 111 genes found in HIP, there were 14 cellular components and 13 biological processes, with synapse organization being the most enriched biological processes. In AMY, only 14 genes were annotated to 5 cellular components and 3 biological processes (Table S7).

Glutamate and GABA are the major excitatory and inhibitory neurotransmitters, and changes in MDD brain connectivity may be associated with altered levels of the major excitatory and inhibitory systems in the brain. Abnormalities in the glutamate and GABA systems have been found in the brains of patients with mood disorders.^[^ ^40^ ^]^ Referring to Loh's histological analysis of synaptic molecules,^[^ ^55^ ^]^ we analyzed the trends of excitatory and inhibitory synaptic genes after CSDS. Compared with RES, glutamate AMPA receptor Gria1 was significantly decreased in the HIP region, Gria2 and Gria3 were significantly decreased in the ISO region, glutamate NMDA receptor Grin2a was down‐regulated in the HIP, while GABA receptors Gabrb1 and Gabra3 were down‐regulated in the Isocortex, and Gabra2 was down‐regulated in the AMY of SUS mice (Figure 4B). Glutamic acid and its homologous receptors play important roles in many nervous system diseases, AMPAR trafficking defects were verified to be involved in the pathogenesis of certain psychiatric and neurodegenerative diseases.^[^ ^56^ ^]^ For example, AMPA receptor GluA1 (Gria1) subunit dysfunction and synaptic plasticity defects were related to schizophrenia, sleep and circadian rhythm disorders,^[^ ^57^ ^]^ The NMDA receptor antagonist ketamine has been shown to produce rapid and sustained antidepressant effects in patients with MDD.^[^ ^58^ ^]^ Glutamic acid and its homologous receptors will be potential therapeutic targets for MDD.^[^ ^59^ ^]^ MDD is associated with various defects in GABAergic transmission,^[^ ^37^ ^]^ and local injection of a GABA(B) receptor antagonist effectively alleviated social avoidance symptoms in SUS mice^[^ ^60^ ^]^.

The study of changes in synaptic structure and function has helped us to gain a deeper understanding of how the brain works during stress. However, due to the location of brain slices, the samples do not include the nucleus accumbens (NAc), ventral tegmental area (VTA) and some areas where c‐Fos signalling is activated by CSDS,^[^ ^61^ ^]^ which are also subject to stress‐induced dysfunction of synaptic plasticity,^[^ ^62^, ^63^, ^64^ ^]^ signal transduction deficits,^[^ ^7^ ^]^ and stress‐induced changes in the strength of connections in specific areas of the brain.^[^ ^65^ ^]^ Furthermore, due to limitations in resolution and current technology, we can only target large areas of tissue, making it challenging to study the neural mechanisms of local tissue structures.

### 3.5 Region‐Region interactions of AMY, HIP, and Isocortex

**FIGURE 5:**
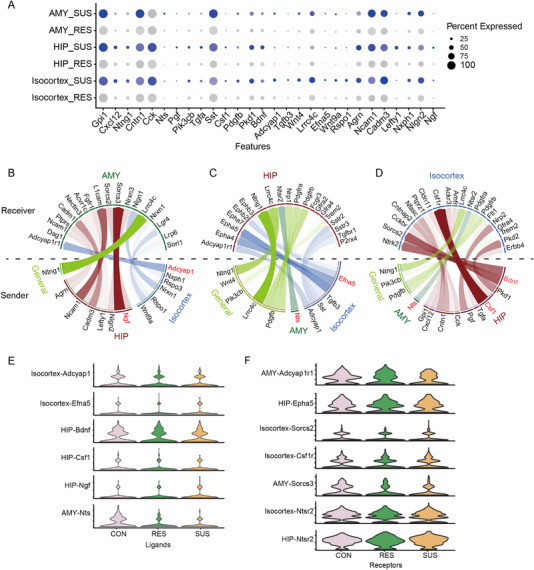
Identification of significant region‐specific and susceptible‐specific ligand‐receptor pairs. A, Top 28 ligand genes predicted by cell‐cell communication analysis in annotated regions (Isocortex, HIP, AMY). Colours represent the level of gene expression (blue shows the significant difference between SUS and RES); sizes indicate the percentage of gene expression. B–D, Circos plot shows significant brain region‐to‐region interactions indicated by ligand‐receptor communication between Isocortex (royal blue), HIP (dark red), and AMY (dark green). Ligand–receptor interactions observed in more than one region are shown in light green. E–F, The expression level of ligand genes E, and related receptor genes F, in Isocortex, HIP and AMY.

To shed light on the interactions among these three brain regions after CSDS, we used Nichenet^[^ ^29^ ^]^ and dsCellNet^[^ ^30^ ^]^ to infer how the communication between brain regions affects gene transcription. We prioritize Isocortex, HIP and AMY regions that play vital roles in dealing with depression and anxiety related emotions and easily form/establish neural circuits associated with stress susceptibility. Our data reveal significant changes in transcription after CSDS,^[^ ^66^, ^67^ ^]^ molecular communication networks between Isocortex, HIP or AMY regions in SUS were established via comparing with RES (Figure 5A–D, Table S8), and ligand–receptor pair expression levels were visualized (Figure 5E,F, Figure S9).

Furthermore, we investigated the potential neural circuits between the Isocortex and the HIP or AMY brain regions by inferring ligand‐receptor interactions. In the Isocortex, ligand genes (such as Adcyap1/PACAP and Efna5) were expressed at high levels (Figure 5A–D). Adcyap1 is involved in widespread regulation of the cellular stress response, Adcyap1 and its cognate PAC1 receptor (Adcyap1r1) locate at stress‐ and anxiety‐associated brain regions,^[^ ^68^ ^]^ and Adcyap1/ Adcyap1r1 pathway plays a potential role in the development of PTSD.^[^ ^69^ ^]^ We also found the signal transduction of Adcyap1r1 projected by Isocortex had an impact on AMY stress and maladaptation. Ephrin‐Eph is a tyrosine kinase receptor signal pathway, participates in cell proliferation, differentiation, migration, tissue remodelling, cell adhesion, and migration,^[^ ^70^ ^]^ as well as regulating synapse formation and neural plasticity. Epha4 knockdown saved depression related phenotype and synaptic defect caused by CUMS in mice.^[^ ^71^ ^]^ EphA7 signal transduction regulated cortical dendritic development and dendritic spine maturity.^[^ ^72^ ^]^ Communication between Efna5 and its receptor Epha4/Epha5/Epha7 may provide a potential treatment for depression.

The HIP‐derived ligands included brain‐derived neurotrophic factor (Bdnf), CSF1, nerve growth factor (Ngf), etc. (Figure 5B–D). Bdnf modulates neuronal morphology and synaptic plasticity via TrkB activation, including long‐term potentiation (LTP). BDNF‐mediated signal transduction is regionally specific in response to stress and antidepressants. When BDNF was bilaterally injected into the dentate gyrus of the hippocampus, it manifestated antidepressant effect in both LH and FST.^[^ ^73^ ^]^ Its receptor Sorcs2 acts as the link between proBDNF/BDNF signal pathway and mental disorders.^[^ ^74^ ^]^ Csf1r was mainly expressed in microglia which would undergo morphological and functional changes after CSDS exposure. Csf1/Csf1r signal transduction involved in the proliferation, activation and neuronal remodelling of microglia induced by CSDS. Csf1/Csf1r plays an important role in neurodegeneration such as demyelinating diseases and Alzheimer's disease (AD).^[^ ^75^ ^]^

The central nervous system (CNS) contains a large amount of Ngf and its receptors, which are involved in neuronal survival, differentiation, proliferation, neuronal plasticity and cognition.^[^ ^76^, ^77^, ^78^ ^]^ Stress leads to changes in Ngf levels in the CNS, increasing susceptibility to diseases associated with mood disorders, including the risk of depression, PTSD and schizophrenia.^[^ ^76^, ^79^ ^]^ Sorcs3 is a growth factor Vps10p domain receptor, localized in postsynaptic density, and involved in protein trafficking between intracellular vesicles and the plasma membrane.^[^ ^80^, ^81^ ^]^ It is associated with the behavioural phenotype of depression, AD and other psychiatric disorders.^[^ ^82^ ^]^ Sorcs3‐deficient mice showed deficits in hippocampal LTD behaviour, especially in the accelerated extinction of fear memory.^[^ ^81^ ^]^ Therefore, Ngf/Sorcs3 may play a potential role in CSDS‐induced synaptic dysfunction in mice.

Additionally, Nts is a key neuromodulator, related to reward, punishment processing, NREM sleep, and hedonic feeding.^[^ ^83^, ^84^ ^]^ Neurotensin plays an emotional role in the basolateral amygdala and regulates the positive and negative signal transduction of the brain in different environments.^[^ ^85^ ^]^ Amygdaloid body is relatively rich in NT immunoreactive elements and NT receptors, and it may modulate the Isocortex/HIP through the ligand‐receptor pair of Nts‐Ntsr2 (Figure 5C,D).

**FIGURE 6:**
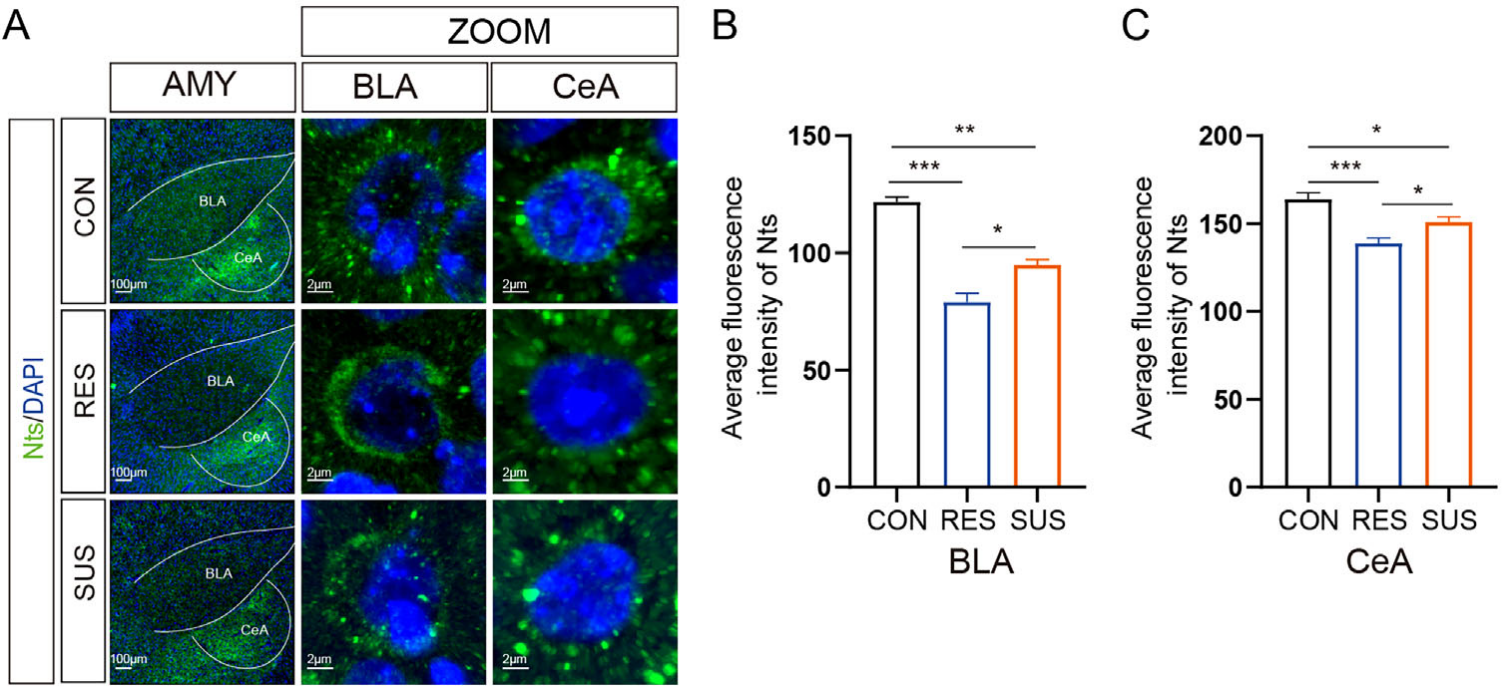
Nts was differentially expressed in AMY exposure to chronic stress. A, Representative image of CON, RES, and SUS mRNA by immunofluorescence (IF). The distribution of fluorescence expression of BLA and CeA Nts gene expression by IF after social defeat. Scale bars: 100 and 2 μm. B–C, Immunofluorescence intensity for Nts was measured. Nts (green, Alexa Fluor 488). *n* = 3 animals per group. Mean ± SEM. *p < 0.05, **p < 0.01; ***p < 0.001 (one‐way ANOVA).

To further determine the potential role of Nts in the stress response, we first examined the expression of Nts in the AMY region after CSDS Immunofluorescence staining showed that SUS mice displayed fluorescence signal of Nts was significantly reduced in both the BLA and CeA regions compared to CON mice (*p* < 0.05, and *p* < 0.01, respectively). Nts was also reduced in RES compared to CON mice (BLA: *p* < 0.001; CeA: *p* < 0.001). These results are consistent with the reduced Nts signalling we observed in the CSDS mouse model (Figure 6A–C). The potential role and mechanisms of Nts signaling in the amygdala following CSDS deserve further exploration.

In sum, we analyzed the receiving or sending signals according to spatial annotation of brain regions and identified several potential neural circuits between the Isocortex and the HIP or AMY regions, which provide clues for the treatment of depression.

## 4 CONCLUSION

In this study, we used the 10× Genomics Visium platform to perform spatial transcriptome analysis of stress‐induced gene expression pattern in the brain in CSDS mice, which provide spatial information to identify comprehensively transcriptional characteristics throughout multiple brain regions of stress‐induced depression in mice.

To study transcriptional changes across brain regions, we visualized gene synchronicity between brain regions using RRHO, and the results displayed a significant overlap between multiple brain regions with downregulated genes in CSDS‐stressed mice, showing strong changes in the gene synchronicity between the Isocortex, AMY, and HIP brain regions with each other, exhibiting consistent downregulation. Furthermore, we used SPOTlight to infer the differences in cell types between CSDS and CON mice, and found that neurons and glial cells have different susceptibility across various brain regions.

A salient feature of our study was the comprehensive analysis of the gene networks of multiple brain regions in SUS and RES mice, overcoming the independence of genes in previous studies. We analyzed eight cortical brain regions and identified the expression profiles of specific brain regions associated with depression induced by CSDS. The SynGO enrichment analysis further revealed that compared with RES, SUS synaptic disorders mainly occurred in Isocortex, HIP, AMY brain regions, and genes involved in synaptic functions were altered at the transcriptional level. Synaptic pathways such as process in the presynapse and postsynapse were altered by CSDS, and trans‐synaptic signalling and synapse organization were significantly enriched mainly in Isocortex and HIP. These changes in synaptic structure and function disrupted neuronal plasticity in brain circuits that control emotion and cognition.

Finally, we investigated the communication between the brain regions of Iscortex, HIP, and AMY in response to CSDS. These data analysis could predict the signal transduction such as Adcyap1‐Adcyap1r1, Bdnf‐Socs2, and Nts‐Ntsr2 that play important roles in the development of CSDS‐induced depression‐like behaviour. We defined the receiving or sending signals according to spatial annotation of brain regions, and identified the potential spatial signal transmission between specific brain regions, which can reflect the long‐distance communication between neurons for determining new neural circuits.

In conclusion, we revealed anatomical location information and spatial gene expression patterns in CSDS model using the spatial transcriptome. This work offers new insights and approaches to explore the heterogeneity of different brain regions following CSDS stress. It also provides insights into the neural mechanisms of stress‐related subregions.

## CONFLICT OF INTEREST STATEMENT

The authors declare no conflicts of interest.

## Supporting information

Supporting Information

Supporting Information

## Data Availability

Raw sequencing data and annotated gene‐barcode matrix are accessible on GEO using the accession number GSE228394. Any other data associated with this work are available from the corresponding authors upon request.
