# Supplementary material for: Spatial transcriptomic analysis of the mouse brain following chronic social defeat stress
Source: Exploration (Beijing). 2023 Oct 13;3(6):20220133. doi: 10.1002/EXP.20220133 (PMC10742195; doi:10.1002/EXP.20220133)
Supplement: Supplementary file 1 — Supporting Information [file EXP2-3-20220133-s001.docx]

Supporting Information

Spatial transcriptomic analysis of the mouse brain following chronic social defeat stress

**Supplementary Figures**


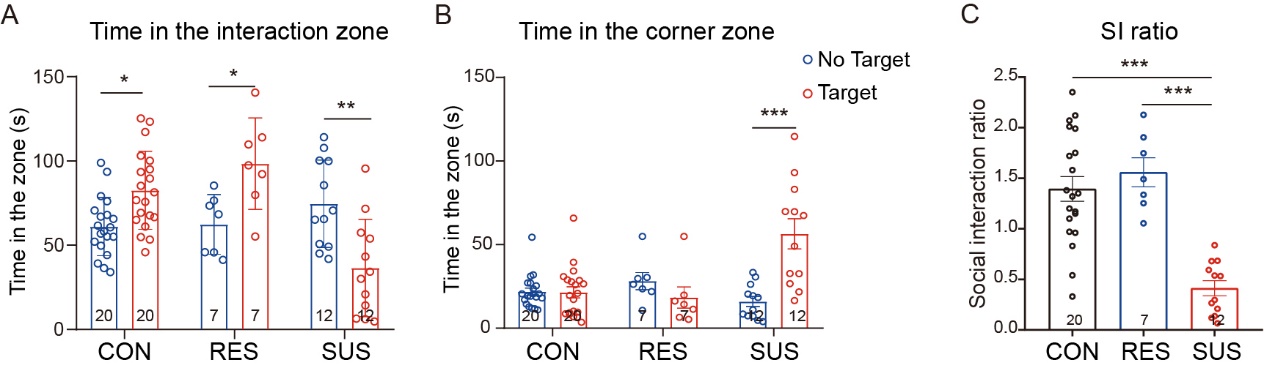


**Figure S1. CSDS-induced depression-like behaviours, Related to Figure 1.** (A, B) The levels of social interaction (A) and social avoidance (B) in CON, RES, and SUS mice respectively. The duration time in the interaction or avoidance zone without and with a CD1 mouse was analyzed. (C) Social avoidance behavior can also be expressed as a social interaction ratio. CON, control; SUS, susceptible; RES, resilient. See also Figure 1. Error bars represent means ± SEM. **p <* 0.05, ***p* < 0.01, ****p* < 0.001 (multivariate ANOVA). The number of mice is shown below each group.


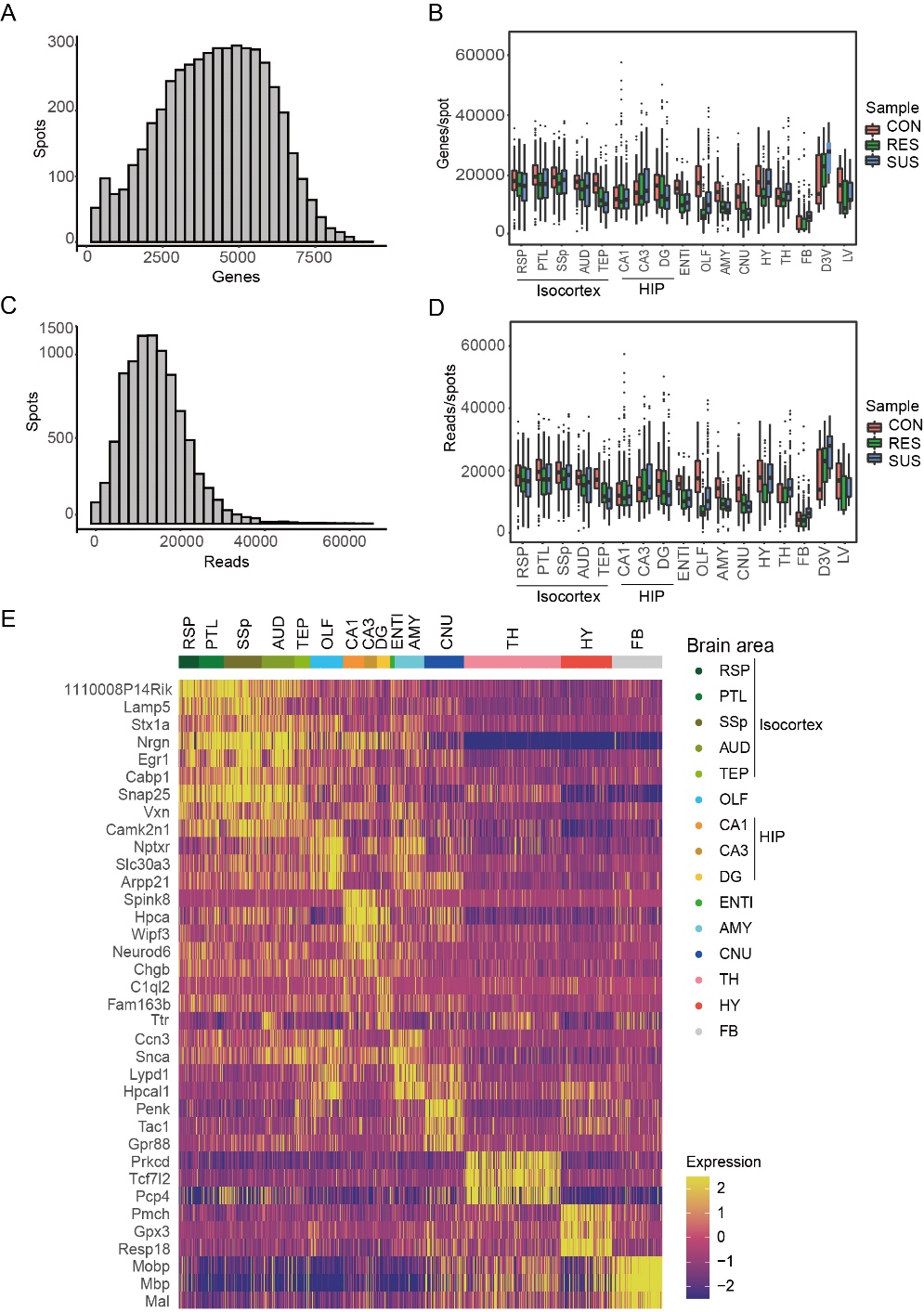


**Figure S2. Summary of sequencing data, Related to Figure 1.** (A) Distribution of the number of genes per spot. (B) Distribution of unique genes per spot in each brain region. (C) Distribution of the number of reads per spot. (D) Distribution of reads per spot, each box representing a brain region. Boxes represent the interquartile range, horizontal lines are medians. (E) Heatmap is the normalized expression of positively enriched signature genes significantly associated with each brain region.


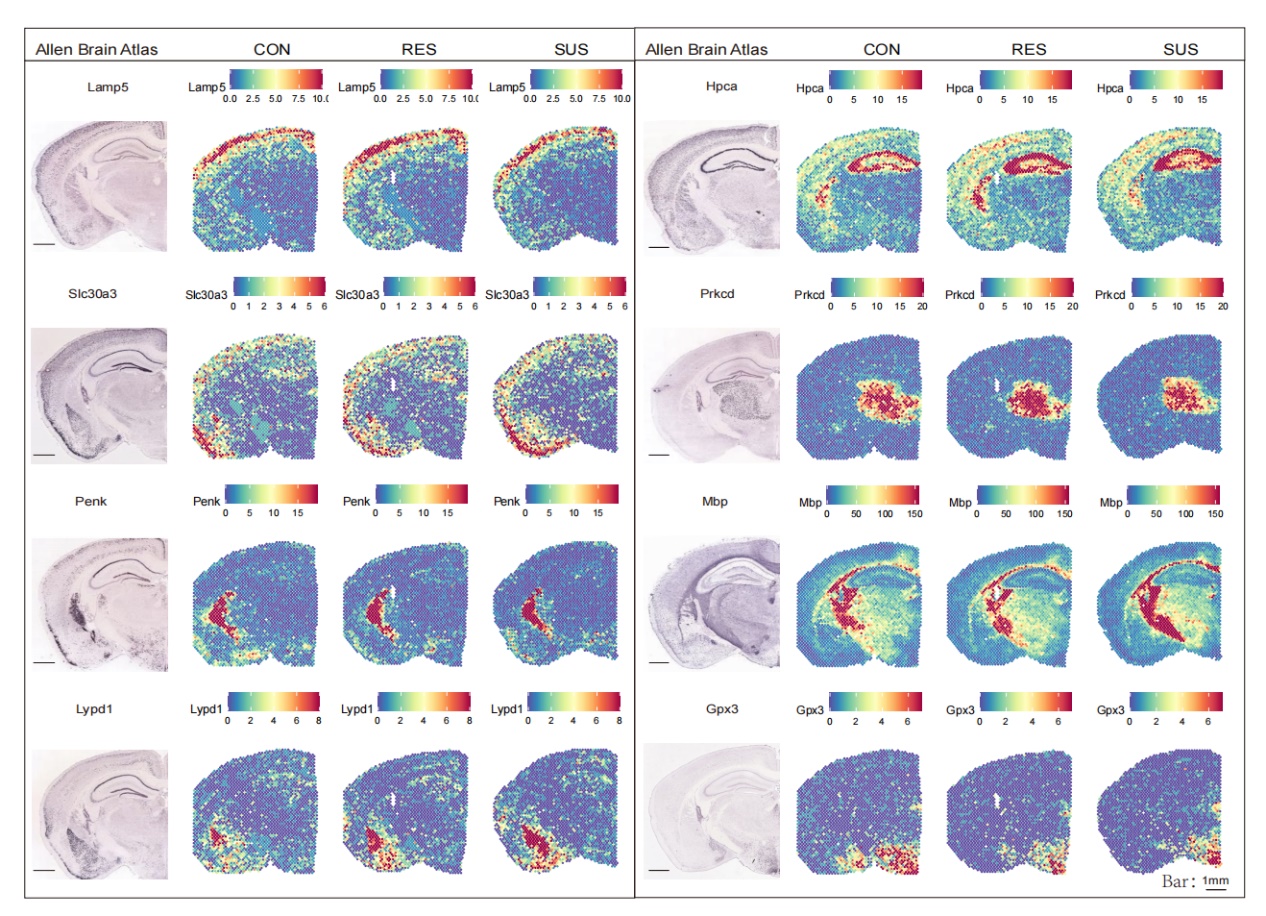


**Figure S3. Visualization of estimated proportions of spatially variable genes on several brain regions, Related to Figure 1.** (A)Marker gene expression obtained by ISH (from the Allen Brain Atlas) in left column, and the right parts are estimated proportions of genes in Brain regions. Lamp5 is a marker gene of Iscortex, Hpca for HIP, SLC30a3 for OLF, Prkcd for TH, Penk for CNU, Mbp for FB, Lypd1 for AMY, Gpx3 for HY. Scale bars represent 1 mm.


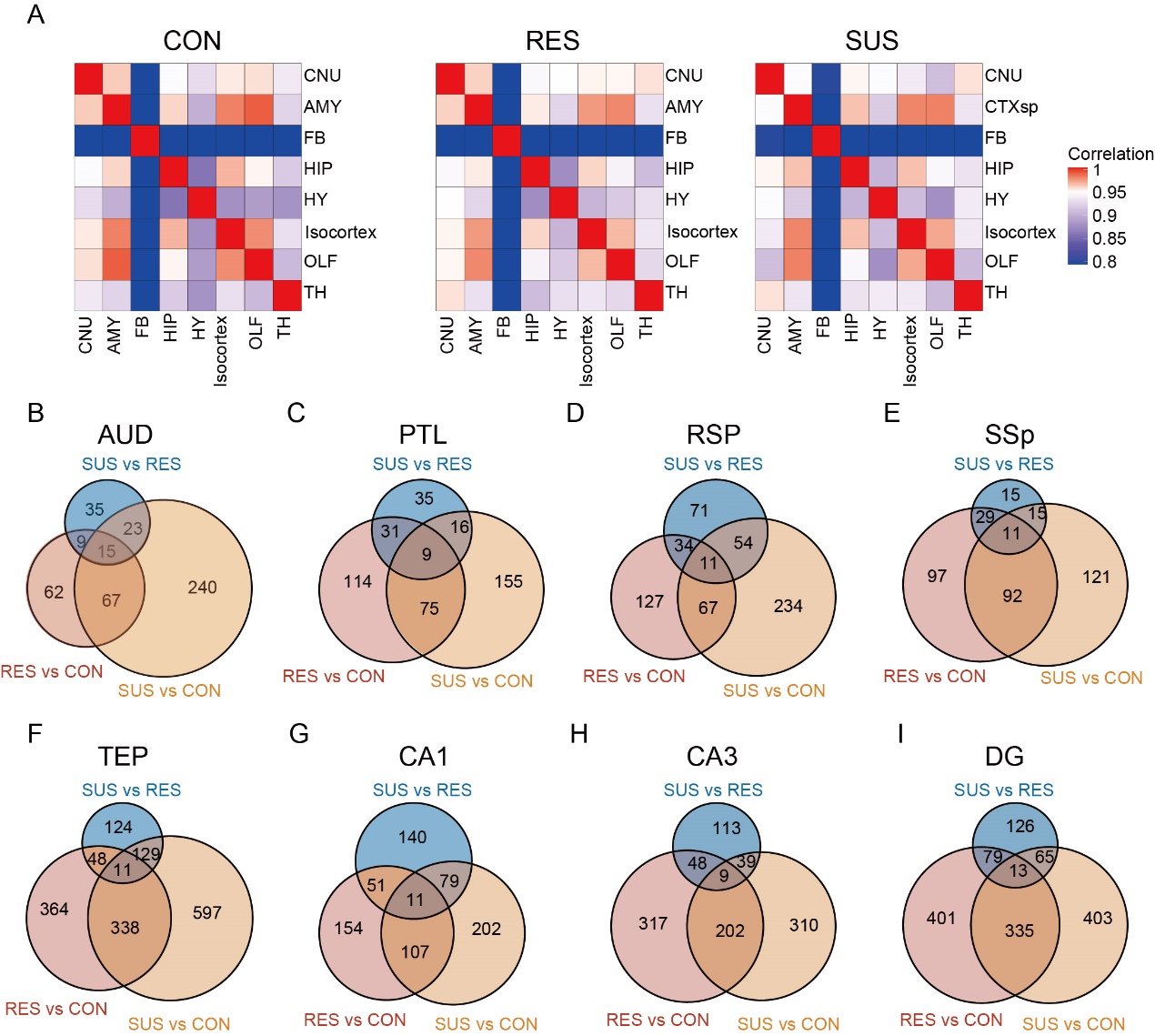


**Figure S4. Gene expression similarity across brain regions, Related to Figure 1.** (A) Gene expression similarity among brain regions in CON, RES and SUS mice. (B-F) Venn diagram showing the overlap of significantly differentially expressed genes in AUD, PTL, RSP, SSp and TEP of Isocortex. (G-I) Venn diagram showing the overlap of numbers of significant differentially expressed genes in CA1, CA3 and DG of HIP.


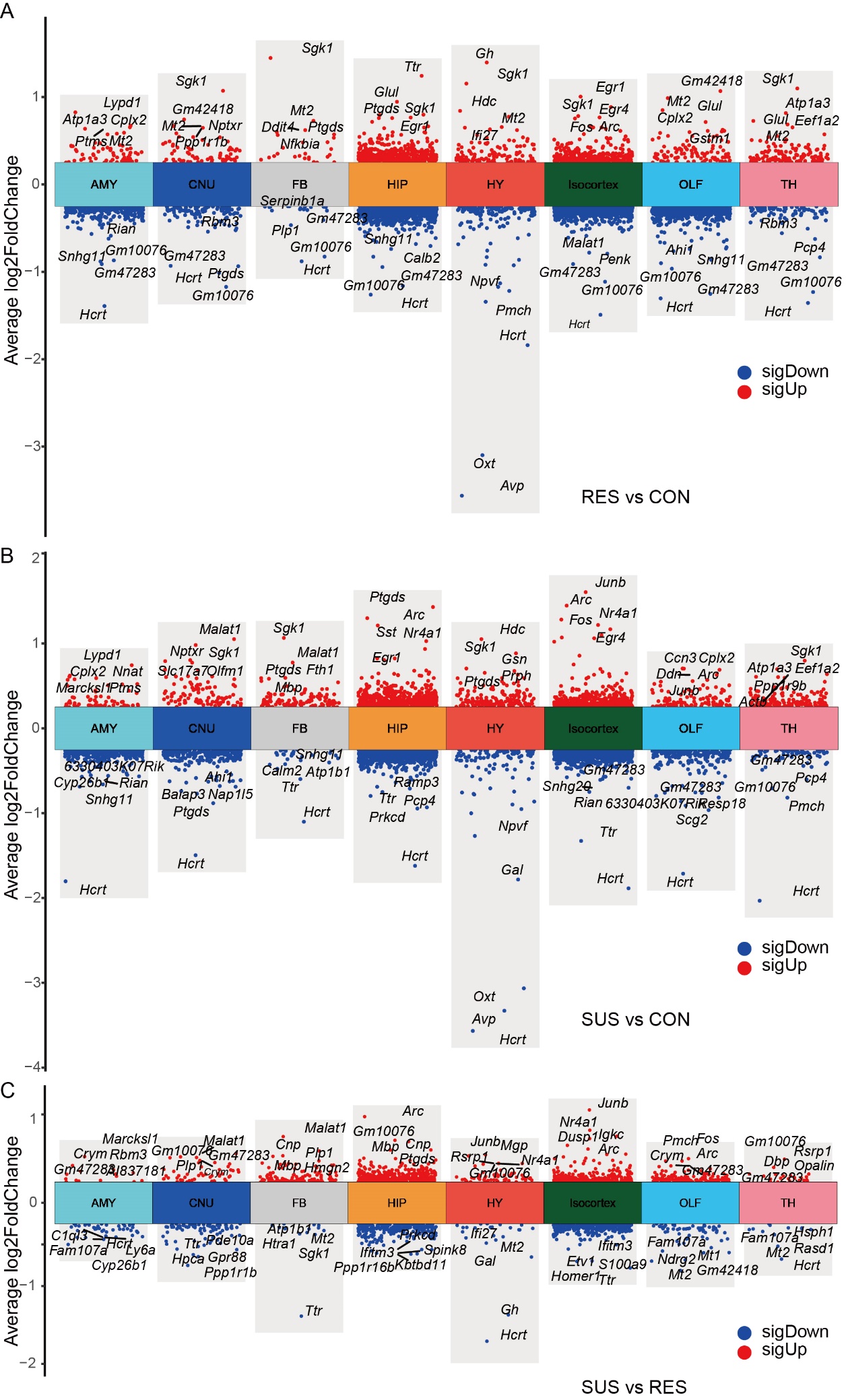


**Figure S5. Up- and down-regulated DEGs in each brain region, Related to Figure 2.** Multi volcano plot showing the fold change of genes (log2 scale) between RES and CON (A), SUS and CON (B), SUS and RES (C) mice. Significantly up-regulated genes (adjusted *p* value < 0.05 & log_2_FC > 0.25) are indicated in red, while significantly down-regulated genes (adjusted *p* value < 0.05 & log_2_FC < 0.25) are indicated in blue.


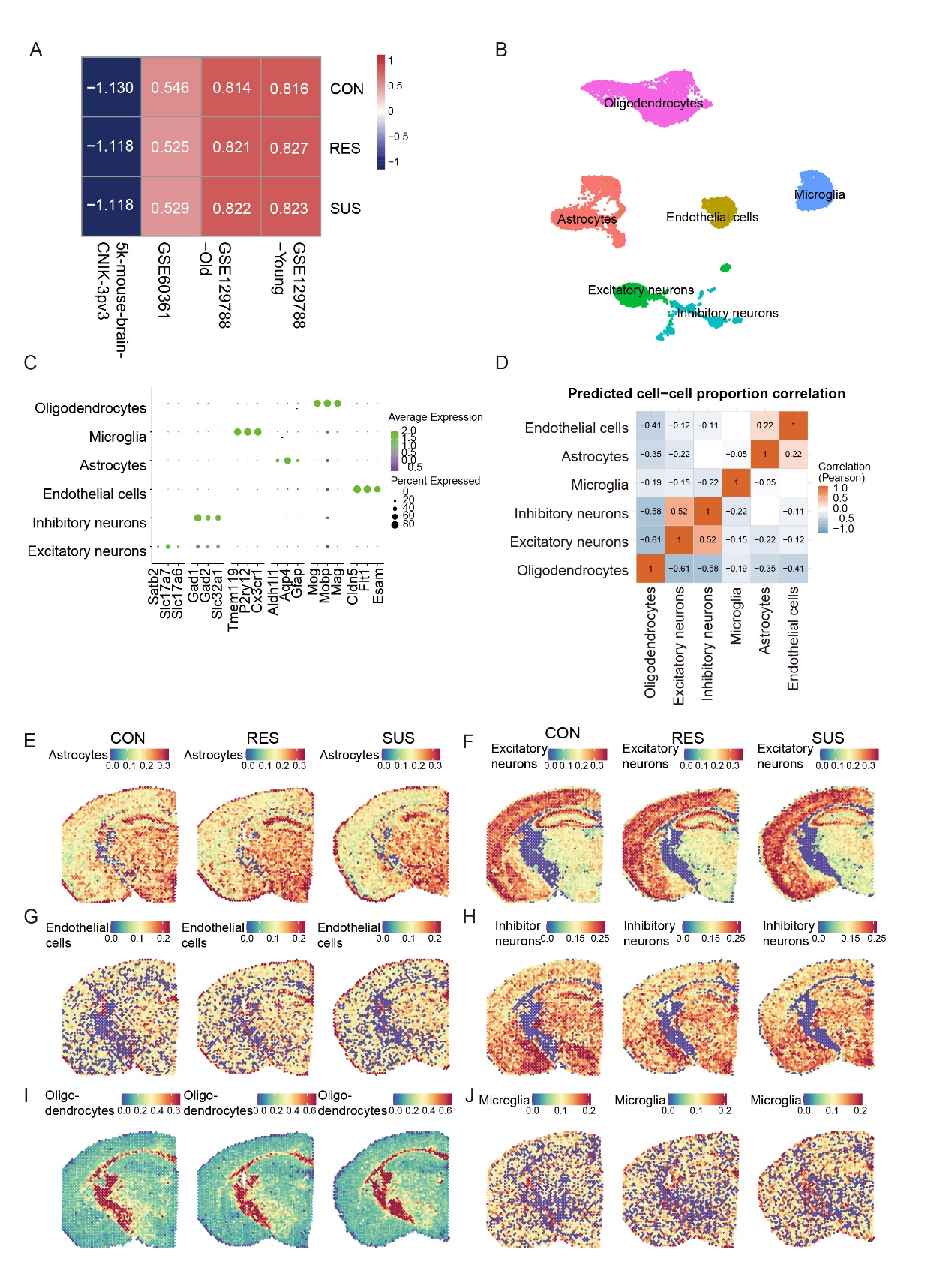


**Figure S6. Graph-based clustering of public scRNA-seq data, related to Figure 3.** (A) Transcriptome correlation between our samples (CON, RES and SUS) and public single-cell RNA-seq data (Mus musculus, C57BL/6, Brian). The scaled Pearson correlation coefficients (r) are indicated. (B) UMAP plot of six selected clusters identified in scRNA-seq data (Ximerakis et al.’s public scRNA-seq data). (C)Dot plot showing selected canonical markers for each cell type. (D) The correlation of predicted cell type proportion deconvoluted using Ximerakis et al.’s public scRNA-seq data. (E-J) Predicted cell type proportion in CON, RES, SUS mice.


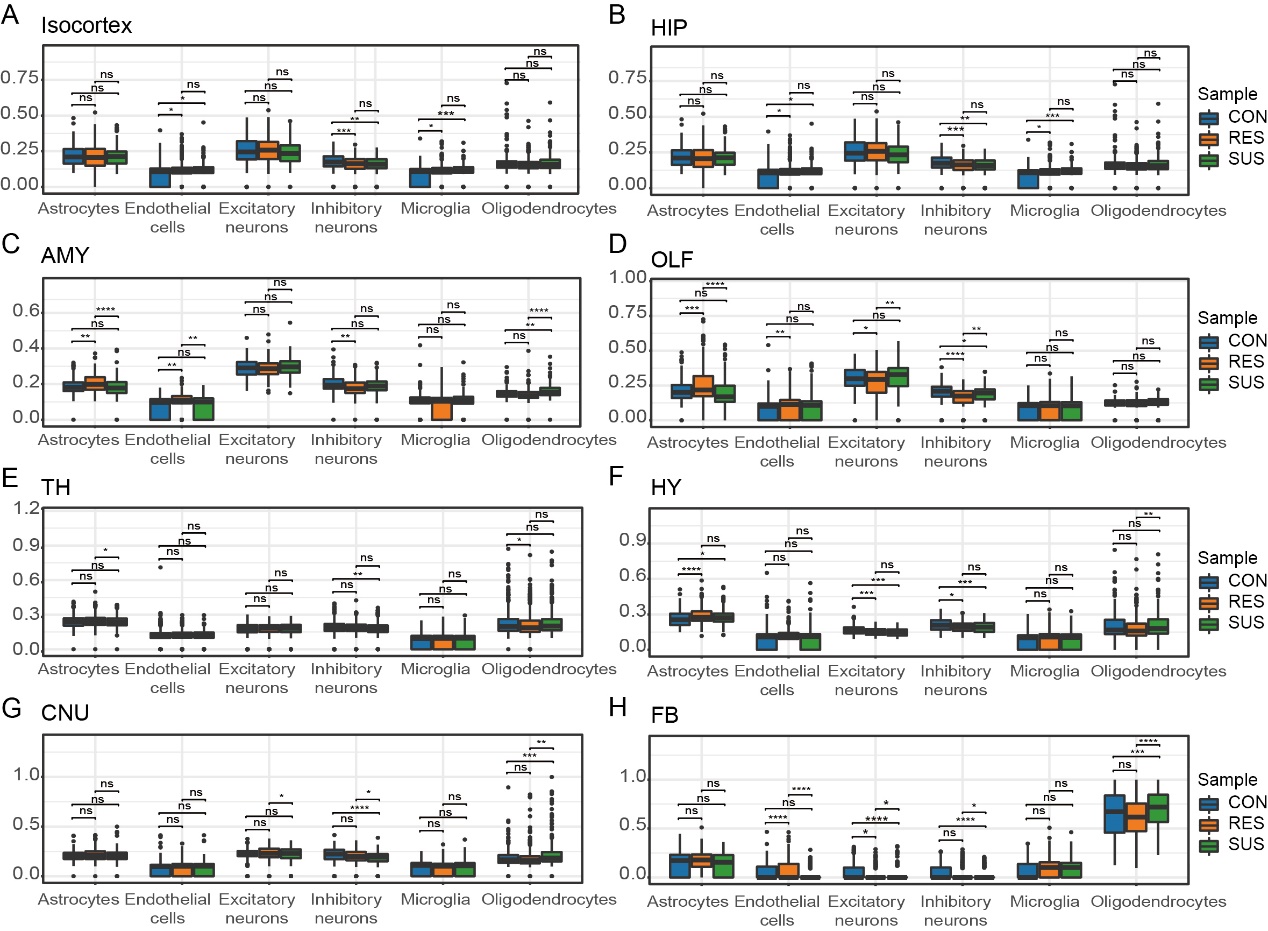


**Figure S**7. Cell type enrichment per region, Related to Figure 3. (A-H) Cell type proportion via comparison among CON, SUS and RES mice across brain regions. *p < 0.05, **p < 0.01, ***p < 0.001, ns: not significant (batch t-test and corrected by Bonferroni test).


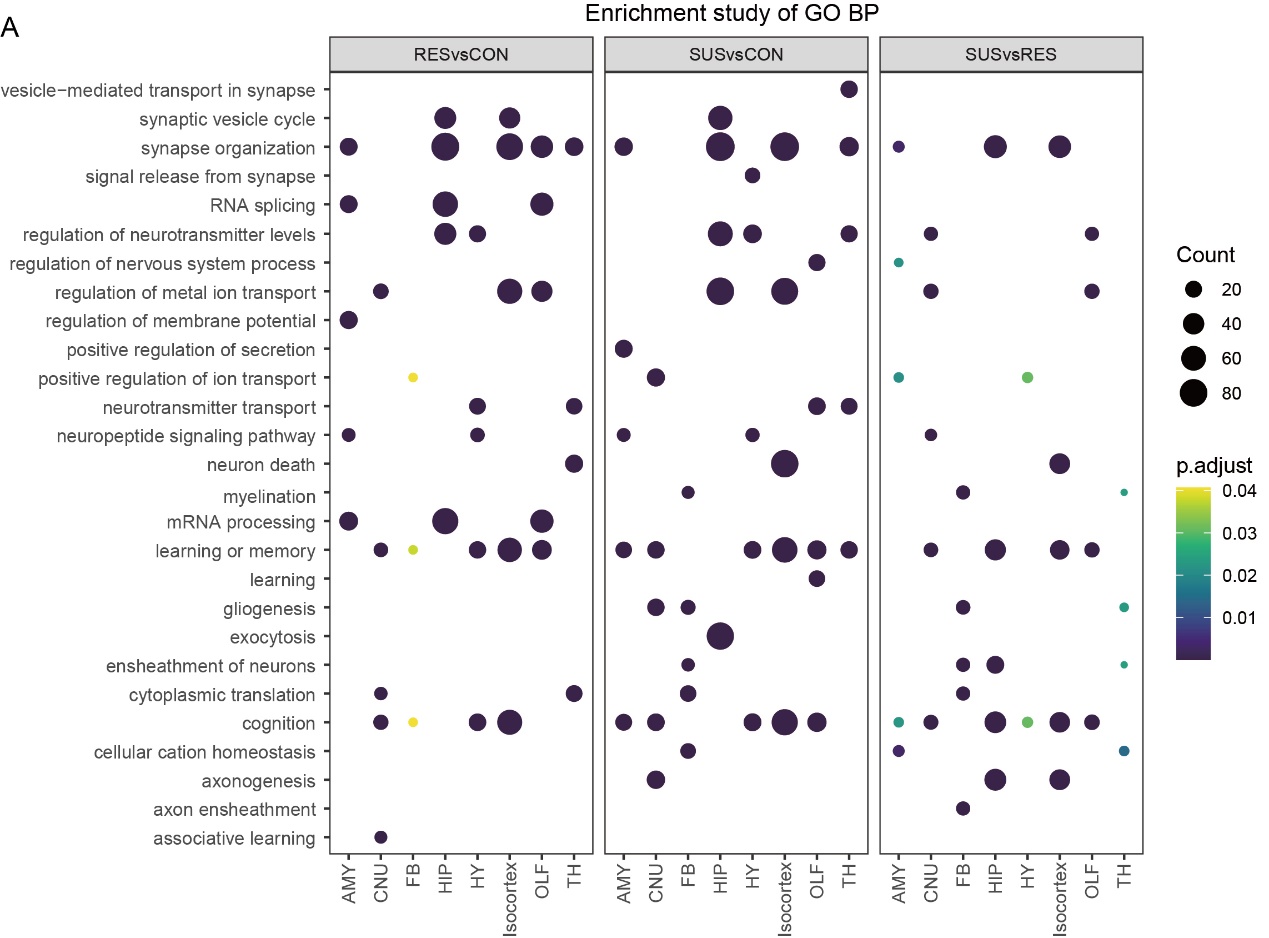


**Figure S**8. GO enrichment analysis of the DEGs identified in comparison of CSDS with control, Related to Figure 4. The bubble chart shows the top 5 representative biological process terms per region enriched in DEGs of RES vs CON, SUS vs CON and SUS vs RES cases. DEGs were assessed with the Seurat FindMarkers function with a log-fold-change threshold of 0.25. Bonferroni-adjusted p-values were used to determine significance at an FDR < 0.05. Analysis was performed using clusterProfiler. The yellow color indicates a relevantly lower significance, blue color indicates a higher significance. The size indicates the number of genes in each term.


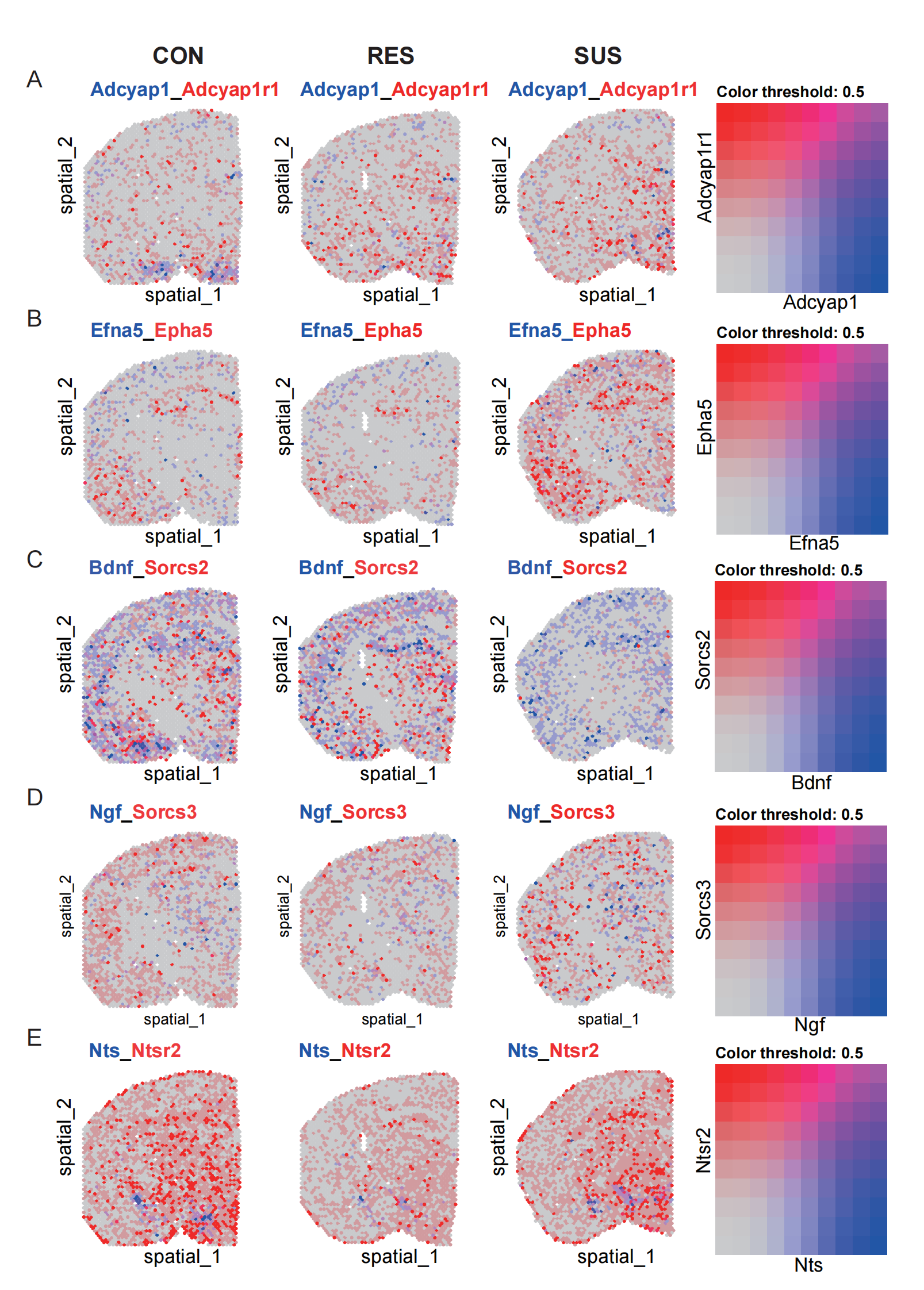


**Figure S9. (A-E)** **Spatial visualization of the expression level of a ligand-receptor pair on the sections, Related to Figure 5.**

**Supplementary Tables**

**Supplementary Table 1.** Spot number of each area in CSDS mice.

**Supplementary Table 2.** Value of RRHO (max–log10(p-value)) between different regions in CSDS mice.

**Supplementary Table 3.** Cell proportion of each cell type in each spot of CON, RES and SUS mice. Each tab contains cell proportion split out by cell type, brain region (Region), Sample(CON, RES and SUS), and the fraction of cells of the specific cell type within the animal of CSDS mice.

**Supplementary Table 4.** Differential gene expression data between CSDS vs CON or SUS vs RES mice. gene name (Gene); original P value (pval); FDR-adjusted P value (padj); log fold change from young to old (log2FC); This file contains 3 worksheet tabs (4-1 for the RES vs CON mice brain, and 4-2 for the SUS vs CON mice brain, 4-3 for the SUS mice vs RES mice brain).

**Supplementary Table 5.** Summary of gene ontology analyses performed on SUS and RES mice DEGs. Hypergeometric test was applied in clusterProfiler. P values are corrected for multiple comparisons using FDR method.

**Supplementary Table 6.** List of SUS dysregulated synaptic genes, compared to RES.

**Supplementary Table 7.** Summary of SynGO enrichment analysis of dysregulation synaptic genes by CSDS (SUS vs RES).

**Supplementary Table 8.** List of the prior interaction weight of the ligand-receptor interaction (SUS vs RES).
